# Undifferentiated round cell sarcoma of the broad ligament

**DOI:** 10.3332/ecancer.2013.303

**Published:** 2013-04-09

**Authors:** Rebeca Diaz-Murillo, Carlos Iglesias-Sanchez, Ignacio Zapardiel

**Affiliations:** Gynecologic Oncology Unit, La Paz University Hospital, Paseo de la Castellana 262, 28040 Madrid, Spain

**Keywords:** sarcoma, broad ligament tumour, round cells tumour, undifferentiated sarcoma

## Abstract

Sarcomas of the broad ligament are very uncommon. To our knowledge, there are no cases published of undifferentiated round cell sarcoma of the broad ligament. Round cell sarcomas are a rare and very aggressive variant, which due to their sensitivity to chemotherapy, have an acceptable prognosis. We report the case of a 27-year-old woman who presented with a pelvic mass with a 7-cm diameter placed on the right broad ligament. After surgery, she was diagnosed with undifferentiated round cell sarcoma of the broad ligament. The patient received adjuvant chemotherapy and radiotherapy, and after 12 years of follow-up, she still remains asymptomatic. Proper differential diagnoses as well as an appropriate adjuvant therapy after surgical treatment seem to be essential to obtain good oncological outcomes in this rare entity.

## Introduction 

In 1957, Gardner [1] defined the characteristics typical of broad ligament tumours, irrespective of their cellular type. This set of tumours occurs extremely rarely, the commonest of them being the benign leiomyoma [2]. Round cell sarcomas include a group of heterogeneous tumours that may encompass Ewing’s sarcoma, liposarcomas, undifferentiated tumours and so on; they are typically more aggressive tumours biologically than the more frequent sarcomas, and they may occur clinically at any age. However, this lack of differentiation means that they are the most chemosensitive of the sarcomas, and are more susceptible and respond better to adjuvant treatment. Therefore, despite having a poor prognosis as a consequence of their biology, they have a high rate of remission following polychemotherapy treatment. Moreover, it is not unusual to resort to radiotherapy for their full treatment. Round cell sarcomas of the broad ligament are an exceptional phenomenon and no published examples of them appear in the literature. However, sarcomatous proliferation in the broad ligament is more frequent in postmenopausal women. To date, only 15 cases of leiomyosarcomas [3] have appeared in the literature, together with one instance of Ewing’s tumour [4] and the other occasional rare instance of undifferentiated tumours not involving round cells. We present a case study of a patient with an undifferentiated round cell sarcoma of the broad ligament that acheived an oncologically satisfactory result.

## Clinical case study

**Figure 1:**
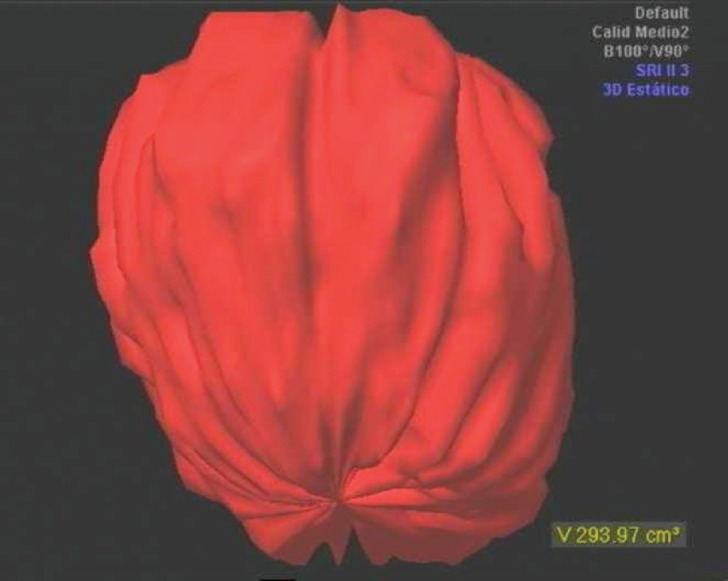
Volumetry with 3D echogram for measurement of the sarcomatous mass.

We present the case of a 27-year-old woman who presented to our department reporting difficulty with urinating over the past fortnight. The patient had no medical or surgical background of interest. The physical examination was normal, although a vaginal echogram revealed a pelvic tumoural mass of mixed echogenicity, 8-cm in diameter and with a volume of 293.97 ml (Figure 1). A diagnostic laparoscopy was carried out within three days, revealing a tumoural mass affecting the right broad ligament. A simple hysterectomy was carried out and the tumour removed. The anatomopathological study indicated that this was a mixed intraligamentary cystic tumoural mass, with profuse proliferation of small-to-medium sized cells with little cytoplasm, vimentin positive, and with areas of necrosis and haemorrhaging, corresponding to an unclassifiable intraligamentary round cell sarcoma measuring 8-cm by 7-cm. The patient then received adjuvant chemotherapy with three cycles of adriamycin (dose 80 mg/m^2^) and cyclophosphamide (dose 500 mg/m^2^). The planned six cycles could not be completed because the patient decided against them. Radiotherapy was combined with the second cycle, using telecobalt therapy combined with curie therapy in the vaginal cuff. Throughout the treatment, the values of analytical tests lay within normal limits. The patient was examined every six months for five years and annually after the sixth year. Follow-up was incident free, and the patient remains asymptomatic 12 years after surgery.

## Discussion 

Round cell sarcomas are a little known phenomenon owing to their low frequency. Having reviewed the literature, we can find no published case of an unclassifiable round cell sarcoma of the broad ligament, which is a further indication of their rarity.

There is no consensus or specific approach published in the literature [5, 6], for the most suitable treatment of other intraligamentary tumours as there is insufficient experience on which to base justified assertions. One approach that appears widely accepted as the preferred procedure, and generally applicable to all sarcomas is surgical treatment, including a hysterectomy and double adnexectomy.

In case of a tumour extending to other parts of the uterine cavity, all the visible tumours should be removed with a view to complete cytoreduction, together with a pelvic and para-aortic lymphadenectomy if tumours of a carcinosarcomatous type are involved. In our case, a hysterectomy with removal of the tumoural mass was carried out, since it was not certain that a malignant tumour was involved. However, following adjuvant treatment, it appears that extension of the surgery was probably not necessary.

It appears that the use of adjuvant chemotherapy and radiotherapy may improve the prognosis, although we do not have sufficient evidence of its use in round cell tumours [7]. Despite this, we opted for both adjuvant treatments together with vaginal cuff brachytherapy.

Demonstrated survival in patients with broad ligament sarcomas ranges from 4 weeks to 33 months, with the best rates being associated with cases in which combined treatment was used [8]. Despite the fact that our patient refused to complete the six cycles of chemotherapy, her follow-up shows that in this case it was sufficient, probably because the tumour was highly localised in the broad ligament and had not infiltrated adjacent structures.

The prognosis for these kinds of tumours in general is uncertain owing to the variability of tumour type. In our case, there was no recurrence after 12 years of follow-up, with the patient remaining asymptomatic, although this is probably due rather to the specific biology of the tumour than to the treatment received, since we know that other round cell tumours in other locations are extraordinarily aggressive despite correct treatment [3, 4].

In conclusion, broad ligament sarcomas are an extraordinarily uncommon phenomenon. A correct differential diagnosis of tumours of the broad ligament and appropriate treatment, preferably involving surgery plus adjuvant chemotherapy and radiotherapy in the case of sarcomatous masses, is fundamental to obtaining adequate oncological results.

**Conflict of Interest:** none
